# Efficacy of percutaneous radiofrequency ablation (RFA) for hepatocellular carcinoma in elderly patients

**DOI:** 10.1186/1471-2318-11-S1-A45

**Published:** 2011-08-24

**Authors:** R Benevento, A Santoriello, G Perna, S Canonico

**Affiliations:** 1 Department of Gerontology, Geriatrics and Metabolic Diseases, Second University of Naples, Italy

## Background

HCC is the most common complication of liver cirrhosis, and the incidence of HCC increases with age in cirrhotic patients. The treatment of HCC in elderly subjects allows for some considerations. Hepatic resection can be safely done in cirrhotic HCC patients aging > 70 years, but the prognosis of these patients is less favorable than < 70 years patients, even when curative resection is achieved.

Liver transplantation is an exceptional measure for elderly patients, because they have a high incidence of concurrent diseases and are usually considered a high-risk group for major surgery. Radiofrequency ablation, alcohol injection and transcatheter arterial chemoembolization are not surgical treatments for HCC that are currently performed in elderly patients.

## Materials and methods

Because few studies have addressed the outcome of RFA in elderly patients with HCC, we performed a retrospective study of 43 elderly patients (age 60-70 years) suffering from a total of 53 HCC treated with percutaneous RFA, from January 2002 to January 2008, in the Department of Gerontology, Geriatrics and Metabolic Diseases, Second University of Naples . The indications for RFA treatment were: HCC consisting of three or fewer nodules, each nodule having a maximum diameter of 30 mm; HCC consisting of a single tumor, regardless of size. In all the patients hepatic function was not Child–Pugh grade C. Survival rates, curative effects, and complications of RFA treatment were evaluated. All of the 53 nodules were percutaneously treated under US guidance with all the patients having conscious sedation or general anaesthesia. Complete ablation of the lesion after RFA treatment was assessed in all the patients using Contrast-enhanced ***ultrasonography***, dynamic CT scans or MRI every 3–4 months during the follow-up period (mean 60 months).

## Results

In thirty-five patients (81%) we obtained complete ablation in one session, in six patients (14%) in two sessions and in two patients (5%) in three sessions. Cumulative overall survival rates estimated at 3 and 5 years were 79% and 67%, respectively. Cumulative disease-free survival rates estimated at 3 and 5 years were 31% and 21%, respectively. No major complication occurred.

## Conclusions

These results suggest that RFA treatment should be addressed proactively even if the elderly HCC patient has concurred diseases. Because the complications from the RFA procedure were fewer, RFA is considered a reliable treatment for HCC in terms of therapeutic efficacy and safety. RFA treatment might be considered above hepatic resection for elderly patients.
